# Post-traumatic ischemia of the gallbladder: a case report

**DOI:** 10.11604/pamj.2025.52.144.49595

**Published:** 2025-12-05

**Authors:** Ahmed Zerhouni, Mehdi Idrissi Bourakkadi, Tarik Souiki, Imane Toughrai, Karim Hassani Ibnmajdoub

**Affiliations:** 1 Laboratory for Advanced Research on Digestive System, University Sidi Mohammed Ben Abdellah, *Faculté de Médecine Pharmacie et Médecine Dentaire, Service de Chirurgie Viscérale, CHU* Hassan II, Fès, Maroc

**Keywords:** Post-traumatic, gallbladder, ischemia, case report

## Abstract

Post-traumatic ischemic injuries of the gallbladder are exceptional, and the incidence of isolated gallbladder injuries is even rarer. We present a case of gallbladder ischemia following abdominal contusion, leading to thrombosis of the cystic artery and complicated by rupture. A 35-year-old man presented to the emergency department three days after a blunt abdominal trauma. He complained of persistent epigastric pain radiating to the back and the right upper quadrant. On arrival, the patient had a slightly elevated temperature, normal blood pressure, and moderate tachycardia. Abdominal examination revealed marked tenderness in the right upper quadrant with signs of localized peritonitis. Laboratory tests showed a C-reactive protein level of 300 mg/L and leukocytosis with predominant neutrophils (15,000/mm^3^). Abdominal computed tomography (CT) revealed moderate ascites in the peri-hepatic space, a thickened gallbladder wall, and a discontinuity of the gallbladder wall at the fundus. The patient underwent an emergency laparotomy through a right subcostal incision. Surgical exploration revealed a completely ischemic gallbladder with a rupture at the fundus and localized bile leakage in the subhepatic space. Complete thrombosis of the cystic artery was identified, with no associated injury to the hepatic parenchyma or vascular structures. A cholecystectomy was performed due to the friable nature of the gallbladder. No bile leak was detected, and the postoperative course was uncomplicated.

## Introduction

Traumatic injuries to the gallbladder are extremely rare, accounting for less than 2% of closed intra-abdominal injuries [1,2]. Isolated gallbladder injuries without hepatic or vascular involvement are even more uncommon due to the gallbladder´s protected anatomical location beneath the liver and behind the ribs [1,3]. When such injuries occur, they are typically associated with high-energy trauma, rib fractures, hepatic lacerations, or vascular injuries [4]. Among gallbladder injuries, post-traumatic ischemia is particularly rare. Ischemia results from compromised blood flow, often due to cystic artery thrombosis [5]. This may progress to gallbladder necrosis and perforation, mimicking acute ischemic cholecystitis. Isolated cystic artery injury without associated hepatic trauma is extremely rare and has been scarcely documented [6,7]. Diagnosing traumatic gallbladder ischemia is challenging due to nonspecific symptoms-abdominal pain, right upper quadrant tenderness, and inflammatory signs-that overlap with acute cholecystitis or hepatic injuries [2,5,8]. Clinical presentation may be delayed. Imaging, especially contrast-enhanced computed tomography (CT), is essential, but early ischemic signs may be subtle [5,9].

## Patient and observation

**Patient information**: a 35-year-old male presented to the emergency department three days after sustaining blunt abdominal trauma in a motor vehicle accident. He complained of persistent epigastric and right upper quadrant pain radiating to the back.

**Clinical results**: physical examination revealed a mild fever (38°C), normal blood pressure, moderate tachycardia, and localized peritonitis in the right upper quadrant.

**Figure 1:**
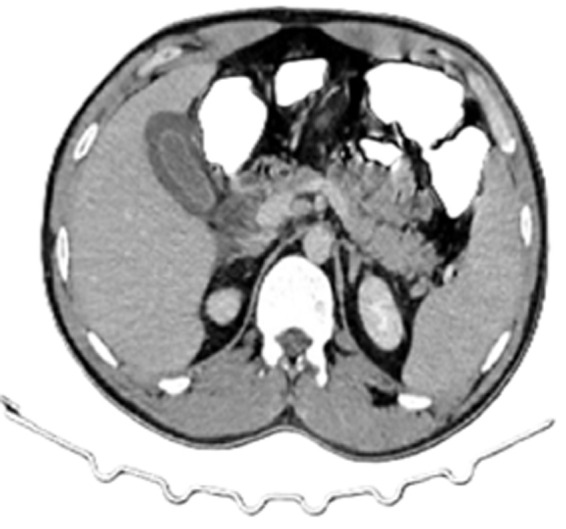
axial computed tomography image demonstrating a thickened gallbladder wall suggestive of ischemic changes

**Figure 2:**
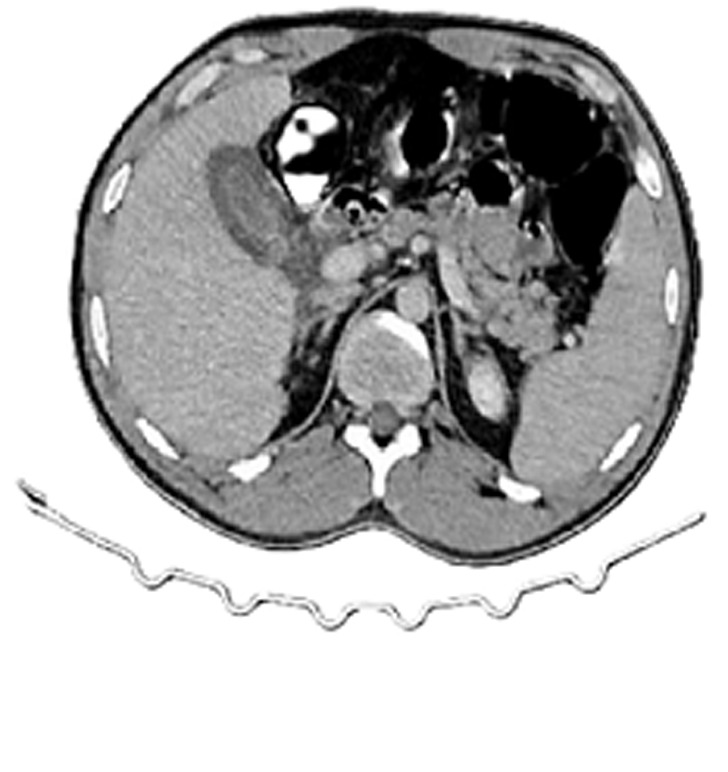
axial computed tomography image showing a gallbladder with diffuse wall thickening, suggestive of ischemia

**Laboratory workup:** showed elevated C-reactive protein (300 mg/L) and neutrophilic leukocytosis (15,000/mm^3^). Computed tomography scan revealed perihepatic ascites, gallbladder wall thickening, and focal wall discontinuity at the fundus (Figure 1, Figure 2).

**Figure 3:**
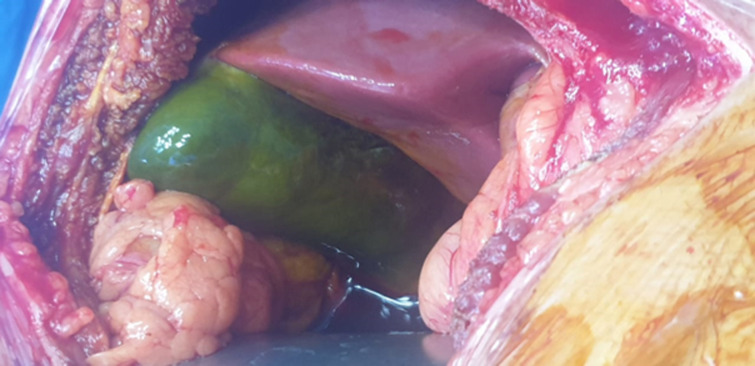
intraoperative view demonstrating a necrotic and ischemic gallbladder

**Figure 4:**
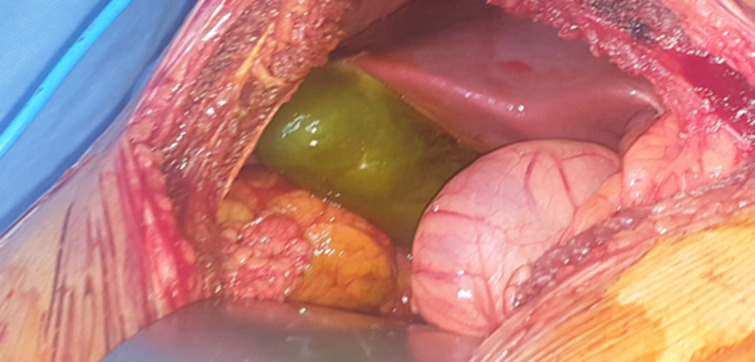
intraoperative image confirms necrosis of the gallbladder

**Figure 5:**
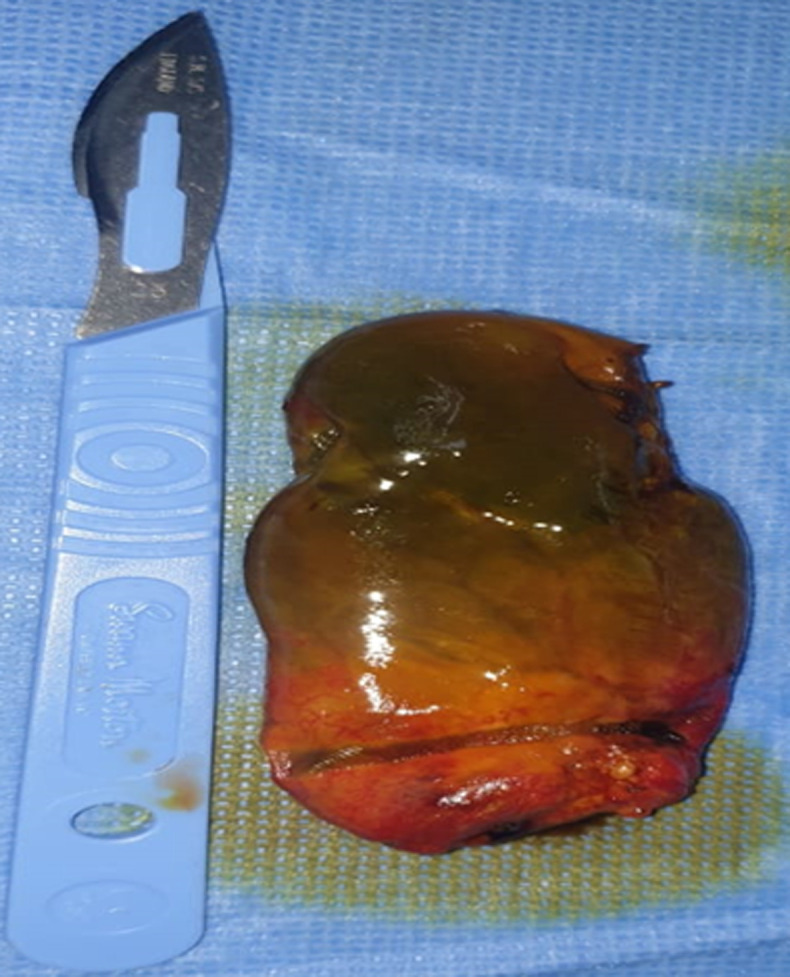
intraoperative photograph revealing a diffusely ischemic and necrotic gallbladder

**Therapeutic intervention and follow-up:** an urgent laparotomy was performed through a right subcostal incision. Intraoperatively, the gallbladder appeared completely ischemic with a fundal rupture and localized bile leak. Complete thrombosis of the cystic artery was confirmed without hepatic or vascular injury. Cholecystectomy was performed. The postoperative course was uneventful (Figure 3, Figure 4, Figure 5).

**Follow-up and results of the intervention:** a return to normal physical activity was observed 7 days after surgery.

**Patient's perspective:** the patient was satisfied in the immediate postoperative period, especially after receiving analgesia 24 hours later.

**Informed consent:** written informed consent for publication of his medical report and for all the interventions was obtained from the patient.

## Discussion

Gallbladder injuries occur in less than 2% of blunt abdominal trauma cases [1]. Most are associated with concomitant hepatic, splenic, or rib injuries [4,10]. Isolated post-traumatic gallbladder ischemia, as in our case, is an exceptional finding [6]. The pathophysiology remains unclear, but cystic artery thrombosis due to direct or indirect trauma appears to be the most likely cause [5,7]. Although the gallbladder benefits from collateral hepatic perfusion, this may not compensate for complete cystic artery occlusion, particularly in hypoperfused patients [2,4]. Symptoms often mimic acute cholecystitis, leading to diagnostic delays. In our case, symptoms appeared three days post-trauma, which is consistent with the progressive ischemic process and eventual rupture [3,6]. Imaging plays a key role. Computed tomography image findings of wall thickening, mural discontinuity, pericholecystic fluid, or lack of enhancement are highly suggestive [5,9]. Ultrasound, though not performed in our case, may show wall thickening and absent Doppler flow in the cystic artery [8]. Surgical treatment is the mainstay. While laparoscopic cholecystectomy is preferred for stable patients, laparotomy is recommended in ruptured or uncertain cases [3,10]. In our case, open surgery was chosen due to suspected perforation and confirmed ischemic necrosis. Early surgical intervention avoids complications such as peritonitis, abscess, or biliary fistula. Our patient recovered uneventfully due to timely diagnosis and management.

## Conclusion

Post-traumatic gallbladder ischemia is a rare but serious condition. Isolated cystic artery thrombosis without hepatic injury is uncommon and difficult to diagnose due to nonspecific symptoms and delayed presentation. Imaging, particularly CT, is critical for early detection. Prompt cholecystectomy remains the treatment of choice. Multidisciplinary awareness of this entity can facilitate early recognition and reduce complications.

## Available online at

https://www.panafrican-med-journal.com//content/article/52/144/full
